# Acute toxicity and genotoxicity of silver nanoparticle in rats

**DOI:** 10.1371/journal.pone.0185554

**Published:** 2017-09-27

**Authors:** Hairuo Wen, Mo Dan, Ying Yang, Jianjun Lyu, Anliang Shao, Xiang Cheng, Liang Chen, Liming Xu

**Affiliations:** 1 National Center for Safety Evaluation of Drugs, National Institutes for Food and Drug Control, Key Laboratory of Beijing for Nonclinical Safety Evaluation Research of Drugs, Beijing, P.R., China; 2 Institute for Medical Device Control, National Institutes for Food and Drug Control, Beijing, P. R., China; 3 Key Lab of Advanced Technologies of Materials, Ministry of Education, School of Materials Science and Engineering, Southwest Jiaotong University, Chengdu, China; Brandeis University, UNITED STATES

## Abstract

**Objective:**

The potential risk of a nanoparticle as a medical application has raised wide concerns, and this study aims to investigate silver nanoparticle (AgNP)-induced acute toxicities, genotoxicities, target organs and the underlying mechanisms.

**Methods:**

*Sprague-Dawley* rats were randomly divided into 4 groups (*n* = 4 each group), and AgNP (containing Ag nanoparticles and released Ag^+^, 5 mg/kg), Ag^+^ (released from the same dose of AgNP, 0.0003 mg/kg), 5% sucrose solution (vechicle control) and cyclophophamide (positive control, 40 mg/kg) were administrated intravenously for 24 h respectively. Clinical signs and body weight of rats were recorded, and the tissues were subsequently collected for biochemical examination, Ag^+^ distribution detection, histopathological examination and genotoxicity assays.

**Results:**

The rank of Ag detected in organs from highest to lowest is lung>spleen>liver>kidney>thymus>heart. Administration of AgNP induced a marked increase of ALT, BUN, TBil and Cre. Histopathological examination results showed that AgNP induced more extensive organ damages in liver, kidneys, thymus, and spleen. Bone marrow micronucleus assay found no statistical significance among groups (*p* > 0.05), but the number of aberration cells and multiple aberration cells were predominately increased from rats dosed with Ag^+^ and AgNP (*p* < 0.01), and more polyploidy cells were generated in the AgNP group (4.3%) compared with control.

**Conclusion:**

Our results indicated that the AgNP accumulated in the immune system organs, and mild irritation was observed in the thymus and spleen of animals treated with AgNP, but not with Ag^+^. The liver and kidneys could be the most affected organs by an acute *i*.*v*. dose of AgNP, and significantly increased chromosome breakage and polyploidy cell rates also implied the potential genotoxicity of AgNP. However, particle-specific toxicities and potential carcinogenic effect remain to be further confirmed in a chronic toxicity study.

## Introduction

Silver nanoparticle (AgNP), having potent broad-spectrum antibacterial properties, strong permeability and little drug resistance, was used to produce a range of antibacterial medical products, such as, toothpaste, gynecologic suppository and wound dressing[1].Potential adverse effects consequentially associated with exposure to AgNPs are of concern. The most prominent characteristic of a metal nanoparticle is that, as a carrier, it could enhance the organ enrichment of ions [2], which also allows its extensive application in targeted cancer treatment and biomedical imaging technology. On the other hand, nanoparticles could take a long period to clear once they accumulated in the organs, and they may have a toxic effect in persistence[3, 4]. Thus, the concerns have been raised on the potential risk of using nanoparticles in medical applications. In recent years, accumulating evidence has shown toxicities induced by AgNP in various *in vitro* experimental models, such as alveolar macrophages [5], neutrophils [6] and also sertoli and granulosa cells [7]. These results need to be confirmed in the *in vivo* system.

Currently, however, the information of AgNPs’ toxicities based on *in vivo* studies is very limited and often controversial. A recent study [8] suggested that short-term oral administration of high doses of AgNP (5 to 100 mg/kg) could significantly increase ROS, ALT, AST, ALP, and lipid hydroperoxide, and cause DNA breakage. By contrast, in a 28-day inhalation toxicity study (1.32× 10^6^ AgNP /cm^3^), no changes on bodyweight, hematology and blood biochemical parameters of *Sprague-Dawley* (SD) rats were observed [9]. Another study also suggested that SD rat oral gavage with up to 36 mg/kg AgNP for 13 weeks showed no obvious change in histopathology, hematology, serum chemistry, micronuclei, and reproductive system parameters [10]. The toxicities that resulted from the different administration routes often varied due to the subsequent distribution patterns. For example, in a single-dose oral administration study [11], the tissue distribution of Ag in the liver, kidneys, and lungs was higher when Ag^+^ was administered compared with AgNP. Whereas intravenously administered AgNP predominantly accumulated in the liver and spleen, and the free Ag^+^ were subsequently released and excreted, and most of which were deposited in the kidneys, lungs, and brain [12]. Therefore, it is crucial to investigate the distribution pattern of AgNPs vs. Ag^+^ and to understand their toxic effects.

Although the genotoxicity of nanoparticles and the underlying mechanisms have been widely studied, most of the results were obtained from cell lines [1, 11, 13–15]. For instance, AgNP induced dose-dependent DNA damage was measured by single cell gel electrophoriesis and cytokinesis blocked micronucleus assay in human lung fibroblas cells. Our previous *in vitro* micronucleus test and golden hamster embryo cells transformation data also suggested the potential genotoxicity and carcinogenicity of AgNP [16]. The persistency of metal nanoparticles in biological systems increases the risk of carcinogenicity, and thus it is important to investigate the tissue distribution, toxicity, and genotoxicity of AgNP *in vivo*.

In this study, SD rat as a popular model was adopted for the *in vivo* bio-distribution and toxicities including genotoxicities triggered by AgNP intravenous administration. In addition, AgNP-released Ag^+^ from the same dose AgNP were also used to compare the toxicological differences between AgNP and AgNP released ion.This research will provide a comprehensive insight about AgNP bio-distribution and AgNP-induced genotoxicity *in vivo*, which will help us have a better understanding of the potential risk of AgNP containing medical devices.

## Materials and methods

### Characterization of AgNP and Ag^+^

**Fig 1:**
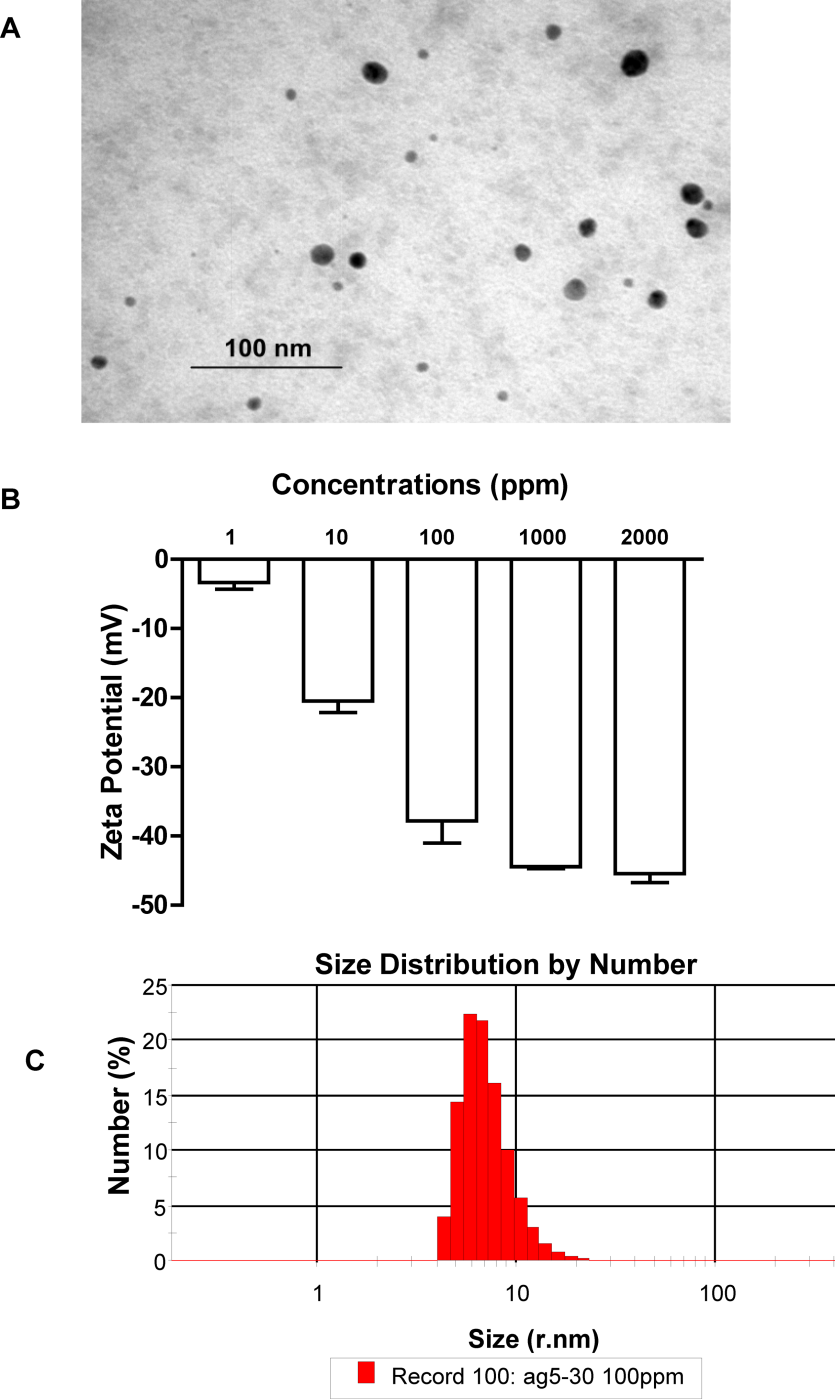
TEM image of AgNPs suspended in 5% sucrose solution.

AgNP solution (2000 μg/mL) was purchased from Nanux (SL1105001, Korea) and the characteristics of AgNP used in this study were described in our previous study[16] The morphology was observed by transmission electron microscopy (TEM, Tecnai G2 20 S-TWIN, America). The size distribution of AgNP suspended in deionized water and the Z-potential were determined using a Malvern Zeta Sizer Nano ZS (Malvern Instruments, Worcestershire, U.K.). AgNP was suspended in a 5% sucrose solution, and the sizes of AgNP were 6.3–629 nm (61.1% ranges from 27.3–106.2 nm. Fig 1). While Ag^+^ were prepared from the supernatant obtained by centrifuging AgNP (1 mg/mL, suspended in a 5% sucrose solution) at 20,000 rpm for 2 h at room temperature after the supernatant was left at 37°C for 24 h, and the concentration of Ag^+^ was detected as 60 ng/mL by atomic absorption spectroscopy (AAS, MKⅡ M6, U.S.).

### Chemicals

Cyclophosphamide monohydrate (Sigma-Aldrich, St Louis, U.S.), colchicine, purity ≥96.0% (Fluka, Buchs, Switzerland), sucrose (Sinopharm Chemical Reagent Co., Ltd, Beijing, China), sodium chloride injection (Shijiazhuang No.4 Pharmaceutical Co., Ltd, Shijiazhuang, China), potassium chloride (Kanto Chemicalco. Inc, Kagaku, Japan), absolute methanol (Beijing Chemical Works, Beijing, China), glacial acetic acid (Beijing Chemical Works, Beijing, China), Giemsa and acridine orange staining buffer (Sinopharm Chemical Reagent Co., Ltd, Beijing, China).

### Ethics statement

All animal experiments and sample collections were performed within the barrier system and a necropsy room at the National Center for Safety Evaluation of Drugs (NCSED). The protocols were approved by the Institutional Animal Care and Use Committee (IACUC) at NCSED and conducted in compliance with China’s national ethical standards to minimize the suffering of animals (see S1 and S2 Files for animal research description).

### Animal grouping and administration

16-week-old wild type specific pathogen free (SPF) female Sprague-Dawley rats (with body weights between 280 g and 310 g) were purchased from Beijing Vital River Laboratory Animal Technology Co., Ltd.(Beijing, China; Animal Quality Certificate No: SCXK(Jing)2007-0001). The rats were housed in polycarbonate cages in a barrier system maintained at 20–25°C with 40–70% relative humidity, a 12 h light-dark cycle, and a room air exchange of 10–20 times per hour. The feeding density was three rats per cage after dosing. Rats had ad libitum access to the certified rodent diet, and sterilized municipal tap water was given ad libitum through water bottles. Each rat was given a unique number and identified by its ear tag and animal number. All rats neither had previous procedures nor abnormal clinical conditions before the study.

SD rats were randomly divided into four groups, including vehicle control, AgNP, Ag^+^, and CPA positive control group (*n* = 4 for each group). 5% sucrose solution was used as vehicle control. CPA was single-dosed *i*.*p*. at 16 h before sacrifice, while the sucrose (vehicle control), AgNP, and Ag^+^ were administered *i*.*v*. at 24 h before sacrifice. The administration doses are 40 mg/kg for CPA, and 0, 5, and 0.0003 mg/kg for AgNP and Ag^+^ respectively. The administration route and doses for the AgNP were chosen to reach the maximum blood concentration possible for genotoxicity. Colchicine was dosed *i*.*p*. at 4 h before sacrifice at 4 mg/kg to maximize the metaphase cells. All animals were anesthetized by CO_2_ inhalation to minimize suffering, then sacrificed by abdominal venesection method.

### Clinical signs and body weight measurement

Clinical signs of animals before and 0–3 h after dosing were carefully observed, including the appearance, activities, hair, possible trauma, feces, and death. Weight change, an important toxicity index for rats, was measured before grouping, dosing, and sacrifice for statistical analysis. No differences were observed within dosing groups as compared to control.

### Biochemical examination

Blood samples for serum biochemical examination (Hitachi 7180 Biochemistry Automatic Analyzer, Hitachi Ltd, Gyeonggi-do, South Korea) were collected through the major veins of the abdominal cavity after anesthetization. Test indexes includes: alanine aminotransferase (ALT), aspartate aminotransferase (AST), alkaline phosphatase (ALP), glucose (GLU), blood urea nitrogen (BUN), glycerin three fat (TG), total cholesterol (CHO), total protein (TP), albumin (ALB), serum calcium (Ca), total bilirubin (TB), serum phosphorus (P), serum chloride (Cl) and creatinine (CRE).

### Ag detection in organs

The heart, lungs, liver, spleen, kidneys, and thymus (*n* = 3–4) were collected and weighed during necropsy. The organ tissues were digested in 6 mL concentrated nitric acid using the microwave digestion system (MARS, CEM, USA). Then, the content of Ag (μg/g) in organs was detected through inductively coupled plasma mass spectrometry (ICP-OES, Optima 5300DV, America).

### Histopathological examination

The heart, lungs, liver, spleen, kidneys, and thymus of animals (*n* = 3) were collected. Tissues were subsequently fixed, dehydrated, paraffin embedded, sliced (about 3 μm thick) and stained with hematoxylin eosin for histological observation under a light microscope.

### Bone marrow micronucleus assay

The unilateral femur of each animal was removed and the bone marrow was washed using fetal bovine serum for harvesting cell suspensions. The cell suspension was centrifuged at 1000 rpm for 5 min, then most of the supernatant was removed. Cells in the remaining supernatant were resuspended, and bone marrow smears were prepared on clean slides (3–4 per animal). Cells were stained by 5% Giemsa to calculate the number of polychromatic erythrocytes (PCE) and normochromatic erythrocytes (NCE) in a total of 200 erythrocytes (ERY) in each animal; 1% acridine orange was used to discriminate and calculate the micronucleated reticulocytes frequencies in 2000 PCEs in each animal.

### Bone marrow chromosome aberration test

Bone marrow cells were harvested from the other femur of each rat for the chromosome abbreviation test. The femur was washed using 5 ml sodium chloride solution. Cell suspension was centrifuged at 1000 rpm for 5 min, and the supernatant was removed subsequently. 0.075M potassium chloride (7 ml) was added to the cells, and incubated at 37°C for 30–60 min for hypotonic treatment. Fixative solution (3 ml, glacial acetic acid: absolute methanol = 1:3) was added to the cells, centrifuged at 1000 rpm for 5 min, and the supernatant was discarded. Then 10 ml of fixative solution was added and cells were incubated at room temperature for 20 min before centrifuging at 1000 rpm for 5 min to remove the supernatant. The fixing was repeated for 2 more times, and fresh fixative solution was added to mix well with the cells. The cells were prepared on clean slides (2–3 slides per animal) using dripping method and dried at room temperature. The slides were stained using 5% Giemsa staining buffer for about 30 min and rinsed with tap water. For each animal, 200 metaphase cells with good dispersion were analyzed to calculate the occurrence rate of cells with chromosomal aberrations.

### Data analysis and statistics

All data are shown as the mean±SD of n values, where n corresponds to the number of rats used. The distribution of all continuous parameters was assessed using Fisher’s exact test. Statistical analysis for Ag concentrations in organs and biochemical examinations calculated by one-way ANOVA, followed by Dunnett’s test for comparisons against control; the micronucleus and chromosome abbreviation assays were analyzed using chi-square test. The figures were prepared using GraphPad Prism 5 for Windows (GraphPad Software, San Diego, CA, USA), and the statistical significance was determined using SPSS (ver.12), as values were considered significantly different when p < 0.05.

## Results

### Clinical signs

No clinical symptom was observed in the vehicle control, Ag^+^, and CPA groups. In contrast, increased breathing rate and decreased activity were observed in all animals in the AgNP group immediately after administration. Brown-red color urine appeared (2 of 4 animals) within 8 h after administrated with AgNP. The brown-red color urine was further investigated in our another study (S1 Text, S1 Table). The red color and massive precipitates were observed in 3 out of 3 urine samples from SD rats on the day administrated with 5 mg/kg AgNP (Nanux) *i*.*v*., and erythrocytes were only clearly observed in one sample under microscope. This phenomenon was disappeared in all animals after 24h. These results together suggested the brownish red colored AgNP in the circulatory system could stain the urine, and the Ag^+^ would acutely excreted from the body through the urinary system. Purpura and congestion were observed around the injection sites of animals administered with AgNP, suggesting vascular irritation and injury. Difficulty in blood collection was experienced in animals administered with Ag^+^ and AgNP.

### Organ distribution of Ag

**Fig 2:**
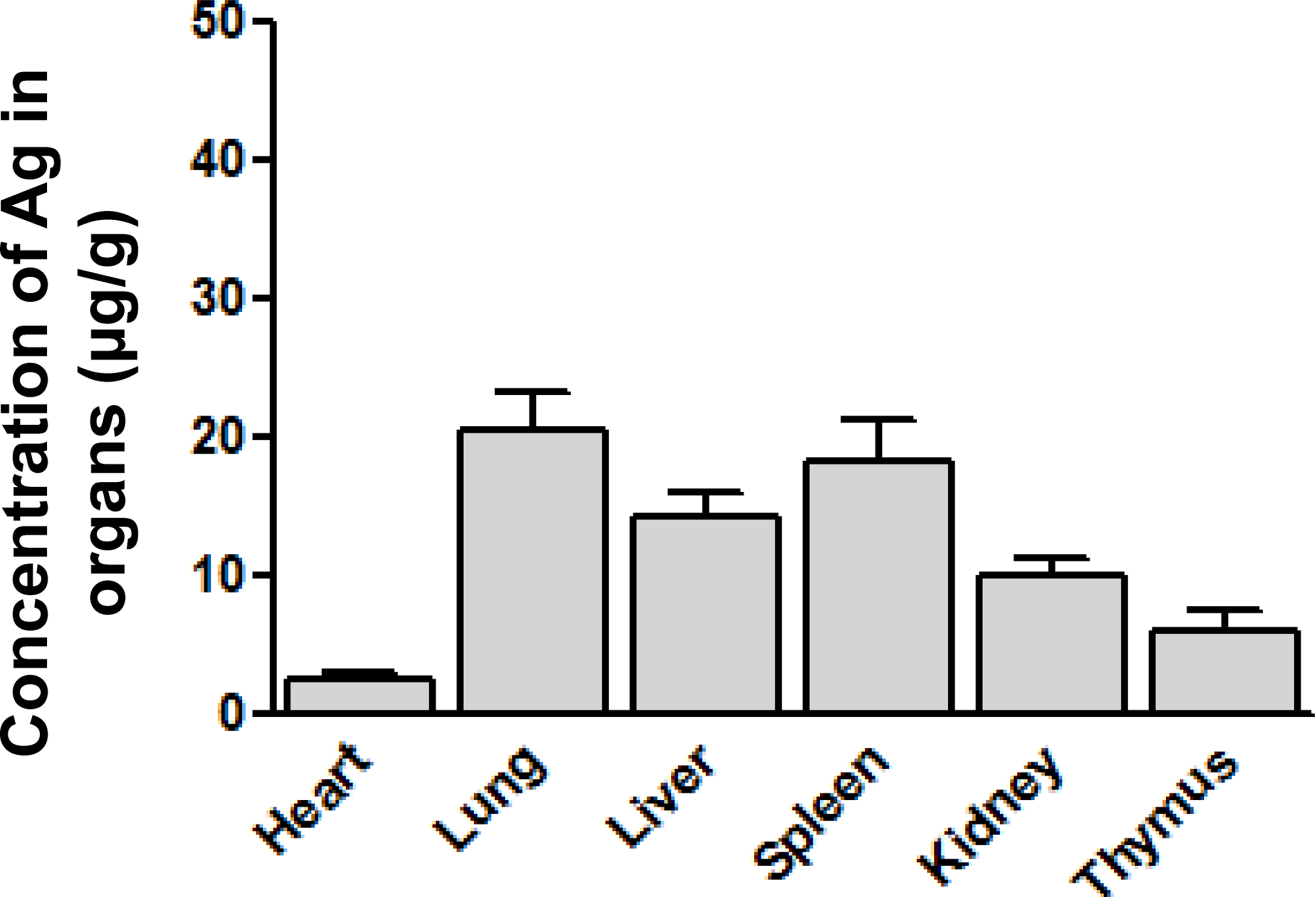
Ag dstribution in organs for SD rats intravenously single-dosed with AgNP (*n* = 3–4). Concentrations of Ag in organs collected from rats administrated with AgNP was analysised, as lung, spleen and liver showed as the major targeted organs.

Bio-distribution of Ag was investigated after a single-dose intravenous administration as shown in Fig 2 (*n* = 3). The rank of Ag detected in organs in AgNP group through ICP-MS from highest to lowest is lung>spleen>liver>kidney>thymus>heart. In contrast, Ag content is undetectable in all organs of animals dosed with the released Ag^+^. Compared with the released Ag^+^ group, the AgNP dosed group demonstrated a different distribution pattern, showing the lungs, spleen, and liver enriched with Ag content.

### Biochemical examination

Intravenous administration of AgNP induced marked increase of ALT, BUN, TBil and Cre, and the reduction of P (Table 1, *n = 3–4*, samples from 4 aminals in the control group and 3 animals in the Ag^+^ group and AgNP group were analyzed), whereas, only decreasing of BUN and P was found in Ag^+^ group. This may be due to the much lower Ag^+^ plasma concentration in rats dosed with Ag^+^.

**Table 1:** Serum biochemical results in SD rats after a single *i*.*v*. dose with AgNP or Ag^+^ (*n* = 3–4).

	Vehicle Control	AgNP	Ag^+^
ALT	442.75±270.74	1540.67±641.49*	159.67±132.88
AST	1098.50±520.57	618.00±1080.80	218.67±284.61
ALP	56.25±72.21	64.33±38.68	54.67±58.05
GLU	6.22±1.65	4.20±1.38	8.92±2.28
BUN	8.85±1.40	11.13±0.40*	6.24±0.97*
TG	0.52±0.36	0.72±0.17	0.67±0.41
CHO	1.56±0.55	1.99±0.68	2.44±0.86
TP	70.40±8.13	72.40±2.99	67.83±8.60
ALB	33.19±3.99	32.82±0.50	31.57±5.35
Ca	0.03±0.05	0±0	0.03±0.06
TB	1.23±0.54	11.73±2.97**	3.30±2.77
P	5.91±0.81	3.46±0.30**	3.16±0.50**
Cl	96.26±3.04	98.50±0.68	98.56±0.48
CRE	26.00±3.16	42.00±1.00**	29.67±2.08

*P<0.05

**P<0.01 One-way Anova.

### Histopathological examination

**Fig 3:**
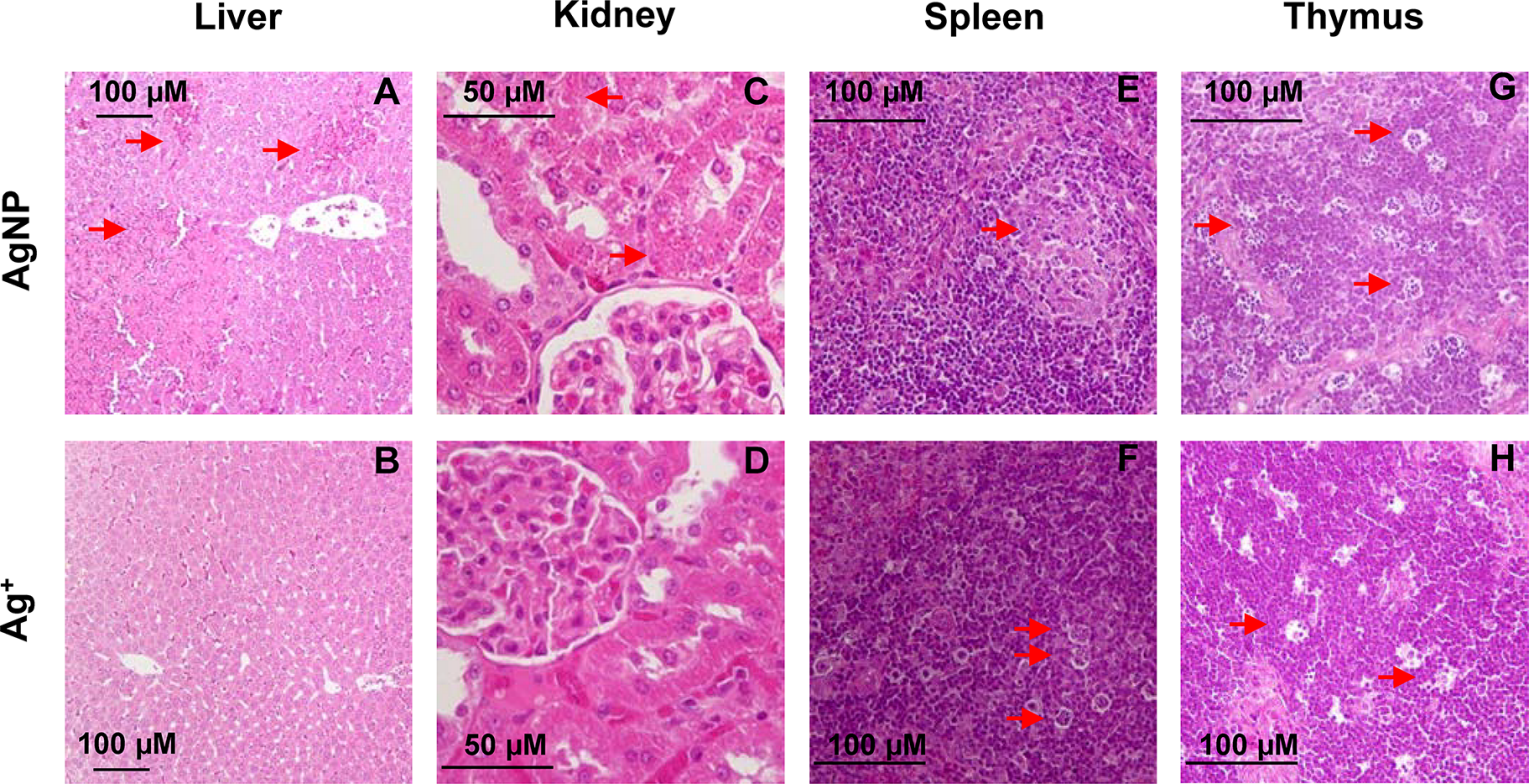
Representative images of histologic changes in liver, kidney, spleen and thymus. All samples prepared from SD rats were hematoxylin and eosin (HE) stained and observed under light microscope (10X-40 X). Moderate multifocal necrosis and hemorrhage of liver cells were observed in animals dosed with AgNP **(A)**, but not in Ag ion group **(B)**. Diffused hyaline degeneration in renal tubular epithelia cell in AgNP group **(C)**, which is absent in Ag ion group **(D)**. In spleen, panel **(E)** showed the necrosis of lymphocytes in AgNP group, while **(F)** exhibited the increased tingible body macrophages caused by Ag ion. Increase of tingible body macrophages in thymus was observed in both groups **(G-H).** Images represent typical samples and changes were indicated using red arrows, *n* = 3.

In contrast to control, AgNP induced more extensive organ damages (Fig 3), such as multifocal liver cell degeneration, necrosis and hemorrhage (3 of 3 animals), diffused hyaline degeneration in renal tubular epithelial cells(3 of 3 animals), increased tangible body macrophages and decreased of lymphocytes in thymus (2 of 3 animals), unifocal lymphocyte necrosis in spleen white pulp (1 of 3 animals). Multifocal increase of tangible body macrophages in spleen (1 of 3 animals) and thymus (2 of 3 animals) were also observed in animals administrated with Ag^+^_._ Critical cell degeneration and necrosis in the liver and kidneys observed in the AgNP group is consistent with the bio-distribution and biochemical results, and due to the high Ag concentrations that accumulated in these organs.

### Genotoxicity

**Fig 4:**
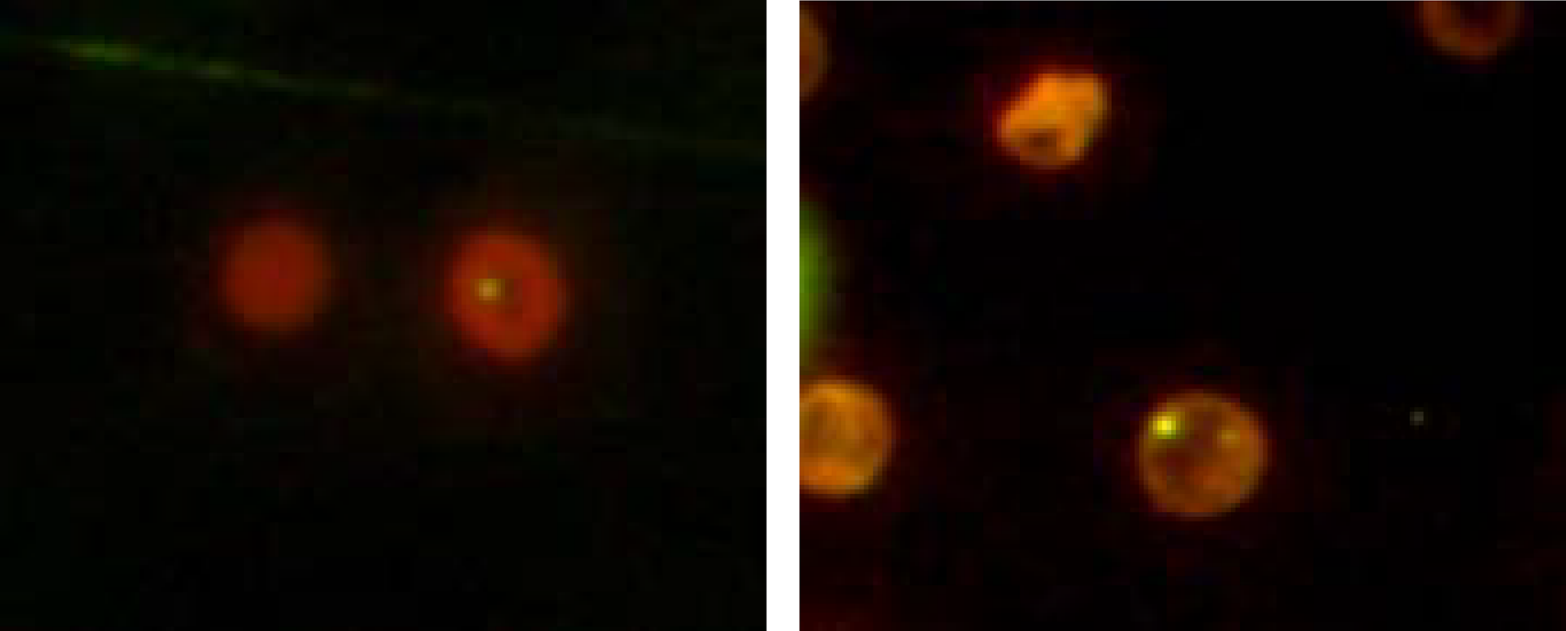
Representative images of polychromatic erythrocytes containing micronucleis induced by AgNP and Ag ions in bone marrow cells of rats. Samples were stained with 0.067% A.O. for 2 min.

The data and representative micronuclei (MN) observed in the bone marrow micronucleus assay are shown (Table 2, Fig 4). No obvious myelosuppression compared to the vehicle control group was observed. Although the MN‰ of samples collected from the Ag^+^ and AgNP groups were relatively higher than those from the vehicle control group (Control: 5.13 ± 0.85‰; AgNP: 12.63 ± 6.93‰; Ag^+^: 9.25 ± 2.99‰ and CPA: 21.13 ± 5.54‰ respectively), no statistical significance was found (*p* > 0.05).

**Table 2:** Average PCE/200 ERY and MnPCE ‰ in SD rat bone marrow cells (*n* = 4, 2000 PCEs per animal).

	PCE/200 ERY	MNPCE ‰
Vehicle Ctl	0.46±0.03	5.13±0.85
AgNP	0.46±0.04	12.63±6.93
Ag^+^	0.48±0.05	9.25±2.99
CPA	0.48±0.02	21.13±5.54

**Fig 5:**
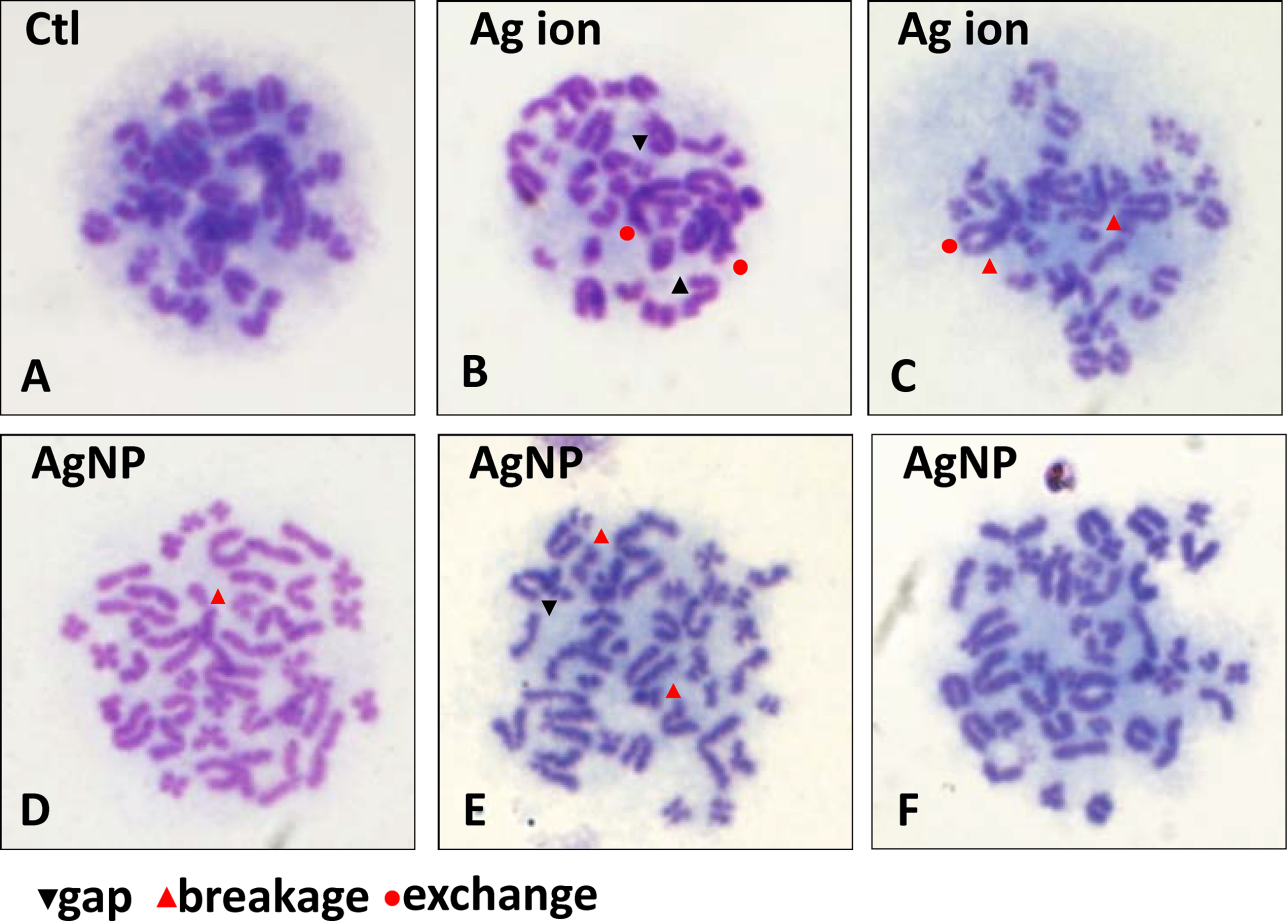
Chromosome aberrations induced by AgNP and Ag ions in SD rat bone marrow cells. All samples were stained with 5% Giemsa for 25 min. **(A)** Vehicle control. Structure aberrations includes gap, breakage and exchange can be observed in both **(B-C)** Ag ion and AgNP treated groups **(D-F)** as indicated in each panel, and a phenomenon with increase of chromosome count in single cell was observed in the latter.

By contrast, a chromosome aberration test revealed the damages caused by Ag^+^ and AgNP in more detail (Table 3, Fig 5). The number of aberration cells (AC) and multiple aberration cells (MAC) were predominately higher in the bone marrow samples from animals dosed with Ag^+^ and AgNP, and the data range were, for AC%, AgNP: 14.3% and Ag^+^: 21.3%; for ACG%, AgNP: 15.1% and Ag^+^: 23.6%; for MAC%, AgNP: 7.1% and Ag^+^: 7.8%. Thesepresented significant differences (*p* < 0.01) when compared with the vehicle control. Although the Ag^+^ generated more structural aberration cells than AgNP (*p* < 0.01), there was no difference between the counts of multiple aberration cells between the AgNP and Ag^+^ groups (*p* > 0.05). The most interesting phenomenon was that more polyploidy cells were generated in the AgNP group (4.3%), while much lesser polyploidy cells can be observed in the Ag^+^ group (0.1%) compared to control.

**Table 3:** Results of SD rat bone marrow chromosome aberration test (*n* = 4, 200 cells per animal).

	Aberration Cell					AC%^2^	ACG%^2^	MAC%^2^	PC%^2^
	ctg/csg^1^	ctb^1^	csb^1^	cte^1^	cse^1^				
**Vehicle Ctl**	6/2	15	25	17	9	5.3	5.4	1.1	1.1
**AgNP**	26/0	42	47	67	24	14.3**	15.1**	7.1**	4.3**
**Ag**^+^	74/12	109	29	91	13	21.3** #	23.6** #	7.8**	0.1*
**CPA**	156/14	200	20	116	44	33.4**	36.4**	12.1**	1.1

*vs. Vehicle Ctl: *p* < 0.05

** vs. Vehicle Ctl: *p* < 0.01

# vs. AgNP: *p* < 0.01

1. ctg/csg: chromatid gap/chromosome gap; ctb/csb: chromatid break/chromosome break; cte/cse: chromatid exchange/chromosome exchange.

2. AC: aberration cell (include gap); ACG: aberration cell (exclude gap); MAC: multiple aberration cell (exclude gap); PC: polyploidy cell.

## Discussion

In this study, acute toxicity and genotoxicity of AgNP (containing Ag nanoparticles and released Ag^+^) or released Ag^+^ (generated from same dose AgNP) after a single *i*.*v*. injection was studied. Biochemical markers and histopathological changes were observed in the liver and kidneys. AgNP accumulated in main immune system organs including the thymus and spleen. Histopathology results also showed that mild irritations were observed in the thymus and spleen only in the AgNP-treated group rather than the Ag^+^-treated group. Furthermore, chromosome breakage and polyploidy cells rates were significantly higher, implying the potential genotoxic and carcinogenic effects caused by AgNP.

The small size of nanoparticles defines their distinct bio-distribution pattern and target organs. The impact of particle size to the AgNP’s toxicity on cell death and cell cycle progression has been reported previously [17, 18]. Particle size plays an important role on the uptake kinetics of NPs in the cells [19]. Our results showed that the Ag were predominantly accumulated in the lungs, spleen, liver, and kidneys of rats dosed with AgNP, suggesting the AgNP transferred and accumulated into specific target organs where they may further generate Ag^+^. However, for the animals dosed with the Ag^+^ alone, the concentration of Ag was undetectable in all organs, probably due to the lower dose of Ag^+^ administration. As reported previously, AgNPs were prone to accumulate in the liver, lungs, and kidneys [20]. After a single *i*.*v*. injection, AgNP distributed into the pulmonary circulation system, and thus predominately accumulated in the lungs, which could potentially lead to chronic lung toxic effects with extended administration period. Due to the short study period, however, we didn’t observe acute lung toxicity induced by AgNP. A recent study [21], nevertheless, has showed evident vascular injury could be induced by a 7 day repeated pulmonary exposure of AgNP (20 nm). The liver and kidneys were the targets of AgNPs in rats via oral administration [22–25], and the TEM data demonstered that the AgNPs were able to penetrate into the liver cells, with the AgNPs tended to bind to proteins around 25–70 kDa. The potential systemic toxicity of AgNPs remains controversial. In general, oral administration of AgNP showed no obvious organ toxicity in subacute toxicity studies [26–28]. However, high concentrations of AgNP in the circulatory system may cause severe toxicities[29]. For instance, intravenously administration of AgNP at 20 mg/kg and above in Wistar rats exhibited significant changes in WBC count, platelet count, hemoglobin, and RBC count, and the levels of liver function enzymes (including ALT, AST, ALP, GGT and TBil) were elevated when 40 mg/kg of AgNP were injected [29]. The maximum blood concentration of AgNP was previously measured as 1 μg/ml with a bioavailability of 4.2% while orally administrated at 10 mg/kg in rats [20]. As such, in our study, the plasma concentration of AgNP after a single *i*.*v*. dose with 5 mg/kg of AgNP is estimated to be about 125 μg/ml. Similarly, the study showed that the toxic effects in the liver and kidneys were observed 48 h after *i*.*v*., and the increased ALT, BUN, TBil and Cre levels implied the appearance of acute liver and kidney injuries in SD rats. Moreover, lymphocyte and macrophage infiltration were revealed as the key observations for AgNP induced morphological changes [24, 30], in addition to cell degeneration, regeneration and necrosis. Nanoparticles introduced inflammation and immune response while interacting with the tissues, which is also a key mechanism of nanotoxicity. Therefore, the mild irritation observed in the spleen and thymus was proposed to be associated with the immune recognition process.

AgNP could enter the human body by digestive tract, respiratory tract, skin or blood vessel, and introduce injuries in liver, kidneys[31], lungs [32]and central nervous system[33]. It was highly persistence in rats and difficult to be excreted from the body, and thus could exerted its toxicity in a chronic mode. The toxicities of AgNP and Ag^+^ at similar concentration levels were compared and different toxiciological mechanisms were exhibited. For example, in a single *i*.*v*. exposure study [34], AgNP demonstrated significant spleen and liver toxicities, while the silver acetate (AgAc) at the same concentration (10 mg/kg) mostly distributed in the kidneys and therefore exhibited a distinct toxic manner. Another study focused on the potential cardiac toxicity of nano materials and demonstrated that both AgNP and Ag^+^ could trigger severe myocardial conditions in mice at dosages above 6 mg/kg. However, AgNP induced a sinus bradycardia and complete atrio-ventricular conduction block, whereas the Ag^+^ led to multifocal ventricular arrhythmias [35].

Whether the toxicities caused by AgNPs were completely attributed to the released ions are yet an elusive question, andour study attempted to discriminate the toxicities produced between AgNP and Ag^+^ liberated immediately from the same concentration of AgNP (estimated to be 0.006% of AgNP). Some previous studies suggested that the Ag^+^, not the nanoparticles, were responsible for the major toxic effects of AgNPs [36, 37]. Most of the toxic effects caused by AgNP were contributed by the dissolved Ag^+^ [38], and the possible mechanisms include activation of lysosomal acid phosphatase activity, disruption of actin cytoskeleton and stimulation of phagocytosis, increase of MXR transport activity, inhibition of Na-K-ATPase, *etc*. An exquisitely designed study[39] ruled out the particle-specific effect of AgNP, since the AgNP showed negligible cytotoxicity to bacteria when synthesized and tested under strict anaerobic conditions to preclude the release of Ag^+^. Recent *in vitr*o studies further pointed out that the toxicity of AgNP is mainly depend on the intracellular release, but not the silver ions liberated in the culture medium [40, 41]. However, Lin et al’s study reported that the lethal bradyarrhythmias could be generated at the presence of AgNP, and further suggested that AgNP was the one contribute to their gross acute toxic effect on myocardial *I*_Na_ and *I*_K1_ channels, as the released Ag^+^ was estimated to be less than 0.02%[35]. In addition, the organism-specific immune response induced by nanoparticles should not be underestimated. Our recent KEGG pathway analysis implied that inflammatory signal pathways in rats can be affected by AgNP but not by ionic Ag alone. It further suggested that inflammatory response may be important for AgNP induced toxicity [42]. The enhanced toxicity produced by AgNP observed in our study, by contrast with the toxicity generated by Ag^+^, is in accordance with both the higher dose and the organ enrichment feature of AgNP.

The genotoxicity of AgNP has been universally evaluated *in vitro* [1, 11]. The Ames test result of AgNP was negative, which might be due to the bacterial being is incapable of endocytosis, whereas the effects on inducing micronuclei formation and DNA breakage was discovered. Few studies have examined the *in vivo* genotoxicity of AgNP so far, and it is innovative to focus on the extra effects of the nanoparticle itself. Our data demonstrated that both AgNP and Ag^+^ produced certain chromosome damages to the bone marrow cells, which were mainly in the form of chromosome or chromatid breakages. Although it is thousands of times less concentrated, such effect is more significant in those injected with Ag^+^, implying Ag^+^, besides the nanoparticle is the fundamental cause of the gap, breakage, and exchange in chromatid and chromosome. The AgNP-induced delay of the cell cycle from G0/G1 to S phase using golden hamster embryo cell model has been previsouly observed[16]. These data altogether suggested that the AgNP might reinforce the chromosomal damage of Ag^+^ together with its effects on the cell cycle [13]. The possible mechanism of AgNP-induced genotoxicity was involved with the interruption of ATP synthesis, subsequent to the disruption of the mitochondrial respiratory chain and excess production of serum reactive oxygen species (ROS) [13].The genetic materials is susceptible to oxidative free radical attacks induced by the metal nanoparticles [2]. Elevated serum ROS can be measured in Wistar rats treated with AgNP, and the single-cell gel electrophoresis data had a significant tail migration [29]. In addition, evidence showed that the AgNP might interact with the DNA directly by disrupting the hydrogen bonding between DNA double strand, and affect its conformation change in calf thymus [43]. Furthermore, the occurrence rate of polyploidy was significantly higher in AgNP group suggested additional genotoxicity induced by AgNP, which was very likely associated with the nanoparticle itself but not the ions. In contrast, the reduced polyploidy rate in Ag^+^ group might be due to the increased chromosome fragmentation. AgNP-induced increased polyploidy rate has been reported previously [44, 45], but has not been highlighted in studies with other metal nanoparticles. Kim et al. showed that AgNP participates in the ROS-induced genotoxicity, which plays important role in mediating DNA and chromosome instability, as well as mitosis inhibition [14]. The increase of polyploidy cells was also suggested to be associated with the activation of the G_2_/M DNA damage checkpoint and ATR/p53/p21 signaling, which can be rescued by an antioxidant treatment [46]. Hence, AgNP-promoted polyploidy formation may relate to the oxidative stress triggered by its interaction with DNA or chromosomes in the cells.

Although we failed to detect the differences of micronucleus rates between the control and AgNP group, a single intravenous exposure to AgNP (5 or 10 mg/kg bw) produced significantly increased micronuclei frequency at 24 h after exposure, and this increase can also be observed 1 and 4 weeks later [3], implying the potential risk of AgNP continuously present in the body. A recent study also showed that SD rats orally administered with AgNP for consecutive 5 days at a range of 5 to 100 mg/kg demonstrated a significant increase of the frequency of micronuclei formation [47]. The negative results of the micronucleus test might relate to the pretreatment of COL and few animal numbers.

## Conclusions

The toxicities of nanoparticle-containing medical devices *in vivo* is currently gain extensive attention, and more knowledge on AgNP is required for safety evaluation and risk management. Taken our data and previous studies together, it is rational to speculate that a lower dose of AgNP with extended exposure period could essentially accumulated in targeted organs and produce chronic toxicity. Furthermore the risk of carcinogenicity may also increase. Major concerns on AgNP’s safety assessment at present are its persistence and disposition in the targeted organs and the subsequent toxicities, which necessitates a chronic toxicity study or carcinogenicity study to follow. The distribution of AgNPs in animals can be identified by, for instance, a recently developed method using gold nanocluster as fluorescence probes [48]. In conclusion, this study sheds light on the underlying distribution, targeted organs and genotoxicities particularly generated by AgNP in SD rat models. The specific toxicities and potential carcinogenic effect induced by nanoparticles need to be further investigated in a chronic toxicity study. For example, a AgNP carinogenic test using C57-ras transgenic mouse model, which are currently performing by our team, might be included.

## Supporting information

S1 Table Urine analysis results of SD rats after single *i*.*v*. dose with AgNP (*n = 3*). (DOCX)

S1 Text Urine collection and analysis for rats dosed with AgNP. (DOCX)

S1 File NC3Rs ARRIVE guidelines checklist-AgNP Wen et al.p1. (TIFF)

S2 File NC3Rs ARRIVE guidelines checklist-AgNP Wen et al.p2. (TIFF)

## Abbreviations

AgNP: silver nanoparticle
CPA: cyclophophamide
COL: colchicine
i.v.: intravenous injection

## References

[1] Xu L, Li X, Takemura T, Hanagata N, Wu G, Chou LL. Genotoxicity and molecular response of silver nanoparticle (NP)-based hydrogel. Journal of nanobiotechnology. 2012;10:16 doi: 10.1186/1477-3155-10-16 ; PubMed Central PMCID: PMC3430588. 22548743 10.1186/1477-3155-10-16 PMC3430588

[2] Manke A, Wang L, Rojanasakul Y. Mechanisms of nanoparticle-induced oxidative stress and toxicity. BioMed research international. 2013;2013:942916 doi: 10.1155/2013/942916 ; PubMed Central PMCID: PMC3762079. 24027766 10.1155/2013/942916 PMC3762079

[3] Dobrzynska MM, Gajowik A, Radzikowska J, Lankoff A, Dusinska M, Kruszewski M. Genotoxicity of silver and titanium dioxide nanoparticles in bone marrow cells of rats in vivo. Toxicology. 2014;315:86–91. doi: 10.1016/j.tox.2013.11.012 . 24321264 10.1016/j.tox.2013.11.012

[4] Mohamed HR. Estimation of TiO(2) nanoparticle-induced genotoxicity persistence and possible chronic gastritis-induction in mice. Food and chemical toxicology: an international journal published for the British Industrial Biological Research Association. 2015;83:76–83. doi: 10.1016/j.fct.2015.05.018 . 26072100 10.1016/j.fct.2015.05.018

[5] Liu YX, Karsai A, Anderson DS, Silva RM, Uyeminami DL, Van Winkle LS, et al Single-Cell Mechanics Provides an Effective Means To Probe in Vivo Interactions between Alveolar Macrophages and Silver Nanoparticles. The journal of physical chemistry B. 2015;119(49):15118–29. doi: 10.1021/acs.jpcb.5b07656 . 26562364 10.1021/acs.jpcb.5b07656

[6] Poirier M, Simard JC, Girard D. Silver nanoparticles of 70 nm and 20 nm affect differently the biology of human neutrophils. Journal of immunotoxicology. 2015:1–11. doi: 10.3109/1547691X.2015.1106622 . 26619040 10.3109/1547691X.2015.1106622

[7] Han JW, Jeong JK, Gurunathan S, Choi YJ, Das J, Kwon DN, et al Male- and female-derived somatic and germ cell-specific toxicity of silver nanoparticles in mouse. Nanotoxicology. 2016;10(3):361–73. doi: 10.3109/17435390.2015.1073396 . 26470004 10.3109/17435390.2015.1073396

[8] Patlolla AK, Hackett D, Tchounwou PB. Silver nanoparticle-induced oxidative stress-dependent toxicity in Sprague-Dawley rats. Molecular and cellular biochemistry. 2015;399(1–2):257–68. doi: 10.1007/s11010-014-2252-7 ; PubMed Central PMCID: PMC4268425. 25355157 10.1007/s11010-014-2252-7 PMC4268425

[9] Ji JH, Jung JH, Kim SS, Yoon JU, Park JD, Choi BS, et al Twenty-eight-day inhalation toxicity study of silver nanoparticles in Sprague-Dawley rats. Inhalation toxicology. 2007;19(10):857–71. doi: 10.1080/08958370701432108 . 17687717 10.1080/08958370701432108

[10] Boudreau MD, Imam MS, Paredes AM, Bryant MS, Cunningham CK, Felton RP, et al Differential Effects of Silver Nanoparticles and Silver Ions on Tissue Accumulation, Distribution, and Toxicity in the Sprague Dawley Rat Following Daily Oral Gavage Administration for 13 Weeks. Toxicological sciences: an official journal of the Society of Toxicology. 2016;150(1):131–60. doi: 10.1093/toxsci/kfv318 . 26732888 10.1093/toxsci/kfv318 PMC5009617

[11] Kim HR, Park YJ, Shin da Y, Oh SM, Chung KH. Appropriate in vitro methods for genotoxicity testing of silver nanoparticles. Environmental health and toxicology. 2013;28:e2013003 doi: 10.5620/eht.2013.28.e2013003 ; PubMed Central PMCID: PMC3577117. 23440978 10.5620/eht.2013.28.e2013003 PMC3577117

[12] Su CK, Liu HT, Hsia SC, Sun YC. Quantitatively profiling the dissolution and redistribution of silver nanoparticles in living rats using a knotted reactor-based differentiation scheme. Analytical chemistry. 2014;86(16):8267–74. doi: 10.1021/ac501691z . 25025651 10.1021/ac501691z

[13] AshaRani PV, Low Kah Mun G, Hande MP, Valiyaveettil S. Cytotoxicity and genotoxicity of silver nanoparticles in human cells. ACS nano. 2009;3(2):279–90. doi: 10.1021/nn800596w . 19236062 10.1021/nn800596w

[14] Kim HR, Kim MJ, Lee SY, Oh SM, Chung KH. Genotoxic effects of silver nanoparticles stimulated by oxidative stress in human normal bronchial epithelial (BEAS-2B) cells. Mutation research. 2011;726(2):129–35. doi: 10.1016/j.mrgentox.2011.08.008 . 21945414 10.1016/j.mrgentox.2011.08.008

[15] Senapati VA, Jain AK, Gupta GS, Pandey AK, Dhawan A. Chromium oxide nanoparticle-induced genotoxicity and p53-dependent apoptosis in human lung alveolar cells. Journal of applied toxicology: JAT. 2015 doi: 10.1002/jat.3174 . 26086747 10.1002/jat.3174

[16] Li X, Xu L, Shao A, Wu G, Hanagata N. Cytotoxic and genotoxic effects of silver nanoparticles on primary Syrian hamster embryo (SHE) cells. Journal of nanoscience and nanotechnology. 2013;13(1):161–70. . 23646712 10.1166/jnn.2013.7077

[17] Rosario F, Hoet P, Santos C, Oliveira H. Death and cell cycle progression are differently conditioned by the AgNP size in osteoblast-like cells. Toxicology. 2016;368–369:103–15. doi: 10.1016/j.tox.2016.08.020 . 27590928 10.1016/j.tox.2016.08.020

[18] Tsiola A, Pitta P, Callol AJ, Kagiorgi M, Kalantzi I, Mylona K, et al The impact of silver nanoparticles on marine plankton dynamics: Dependence on coating, size and concentration. Sci Total Environ. 2017;601–602:1838–48. doi: 10.1016/j.scitotenv.2017.06.042 . 28628993 10.1016/j.scitotenv.2017.06.042

[19] Kettler K, Giannakou C, de Jong WH, Hendriks AJ, Krystek P. Uptake of silver nanoparticles by monocytic THP-1 cells depends on particle size and presence of serum proteins. J Nanopart Res. 2016;18(9):286 doi: 10.1007/s11051-016-3595-7 ; PubMed Central PMCID: PMCPMC5034003. 27774037 10.1007/s11051-016-3595-7 PMC5034003

[20] Park K, Park EJ, Chun IK, Choi K, Lee SH, Yoon J, et al Bioavailability and toxicokinetics of citrate-coated silver nanoparticles in rats. Archives of pharmacal research. 2011;34(1):153–8. doi: 10.1007/s12272-011-0118-z . 21468927 10.1007/s12272-011-0118-z

[21] Holland NA, Thompson LC, Vidanapathirana AK, Urankar RN, Lust RM, Fennell TR, et al Impact of pulmonary exposure to gold core silver nanoparticles of different size and capping agents on cardiovascular injury. Particle and fibre toxicology. 2016;13(1):48 doi: 10.1186/s12989-016-0159-z ; PubMed Central PMCID: PMCPMC4997661. 27558113 10.1186/s12989-016-0159-z PMC4997661

[22] Jimenez-Lamana J, Laborda F, Bolea E, Abad-Alvaro I, Castillo JR, Bianga J, et al An insight into silver nanoparticles bioavailability in rats. Metallomics: integrated biometal science. 2014;6(12):2242–9. doi: 10.1039/c4mt00200h . 25363792 10.1039/c4mt00200h

[23] Park K. Toxicokinetic differences and toxicities of silver nanoparticles and silver ions in rats after single oral administration. Journal of toxicology and environmental health Part A. 2013;76(22):1246–60. doi: 10.1080/15287394.2013.849635 . 24283396 10.1080/15287394.2013.849635

[24] Yun JW, Kim SH, You JR, Kim WH, Jang JJ, Min SK, et al Comparative toxicity of silicon dioxide, silver and iron oxide nanoparticles after repeated oral administration to rats. Journal of applied toxicology: JAT. 2015;35(6):681–93. doi: 10.1002/jat.3125 . 25752675 10.1002/jat.3125

[25] Loeschner K, Hadrup N, Qvortrup K, Larsen A, Gao X, Vogel U, et al Distribution of silver in rats following 28 days of repeated oral exposure to silver nanoparticles or silver acetate. Particle and fibre toxicology. 2011;8:18 doi: 10.1186/1743-8977-8-18 ; PubMed Central PMCID: PMC3123173. 21631937 10.1186/1743-8977-8-18 PMC3123173

[26] Hong JS, Kim S, Lee SH, Jo E, Lee B, Yoon J, et al Combined repeated-dose toxicity study of silver nanoparticles with the reproduction/developmental toxicity screening test. Nanotoxicology. 2014;8(4):349–62. doi: 10.3109/17435390.2013.780108 . 23432083 10.3109/17435390.2013.780108

[27] Kulthong K, Maniratanachote R, Kobayashi Y, Fukami T, Yokoi T. Effects of silver nanoparticles on rat hepatic cytochrome P450 enzyme activity. Xenobiotica; the fate of foreign compounds in biological systems. 2012;42(9):854–62. doi: 10.3109/00498254.2012.670312 . 22458323 10.3109/00498254.2012.670312

[28] Smock KJ, Schmidt RL, Hadlock G, Stoddard G, Grainger DW, Munger MA. Assessment of orally dosed commercial silver nanoparticles on human ex vivo platelet aggregation. Nanotoxicology. 2014;8(3):328–33. doi: 10.3109/17435390.2013.788749 . 23517080 10.3109/17435390.2013.788749

[29] Tiwari DK, Jin T, Behari J. Dose-dependent in-vivo toxicity assessment of silver nanoparticle in Wistar rats. Toxicology mechanisms and methods. 2011;21(1):13–24. doi: 10.3109/15376516.2010.529184 . 21080782 10.3109/15376516.2010.529184

[30] Chuang HC, Hsiao TC, Wu CK, Chang HH, Lee CH, Chang CC, et al Allergenicity and toxicology of inhaled silver nanoparticles in allergen-provocation mice models. International journal of nanomedicine. 2013;8:4495–506. doi: 10.2147/IJN.S52239 ; PubMed Central PMCID: PMC3841295. 24285922 10.2147/IJN.S52239 PMC3841295

[31] Anwar MF, Yadav D, Rastogi S, Arora I, Khar RK, Chander J, et al Modulation of liver and kidney toxicity by herb Withania somnifera for silver nanoparticles: a novel approach for harmonizing between safety and use of nanoparticles. Protoplasma. 2015;252(2):547–58. 10.1007/s00709-014-0701-5. 25248758. doi: 10.1007/s00709-014-0701-5 25248758 10.1007/s00709-014-0701-5

[32] Zhai HJ, Sun DW, Wang HS. Catalytic properties of silica/silver nanocomposites. Journal of nanoscience and nanotechnology. 2006;6(7):1968–72. . 17025110 10.1166/jnn.2006.320

[33] Xu L, Shao A, Zhao Y, Wang Z, Zhang C, Sun Y, et al Neurotoxicity of Silver Nanoparticles in Rat Brain After Intragastric Exposure. Journal of nanoscience and nanotechnology. 2015;15(6):4215–23. . 26369032 10.1166/jnn.2015.9612

[34] Recordati C, De Maglie M, Bianchessi S, Argentiere S, Cella C, Mattiello S, et al Tissue distribution and acute toxicity of silver after single intravenous administration in mice: nano-specific and size-dependent effects. Particle and fibre toxicology. 2016;13:12 doi: 10.1186/s12989-016-0124-x ; PubMed Central PMCID: PMCPMC4772516. 26926244 10.1186/s12989-016-0124-x PMC4772516

[35] Lin CX, Yang SY, Gu JL, Meng J, Xu HY, Cao JM. The acute toxic effects of silver nanoparticles on myocardial transmembrane potential, INa and IK1 channels and heart rhythm in mice. Nanotoxicology. 2017:1–11. doi: 10.1080/17435390.2017.1367047 . 28830271 10.1080/17435390.2017.1367047

[36] Sakamoto M, Ha JY, Yoneshima S, Kataoka C, Tatsuta H, Kashiwada S. Free silver ion as the main cause of acute and chronic toxicity of silver nanoparticles to cladocerans. Archives of environmental contamination and toxicology. 2015;68(3):500–9. doi: 10.1007/s00244-014-0091-x . 25352442 10.1007/s00244-014-0091-x

[37] Hadrup N, Loeschner K, Bergstrom A, Wilcks A, Gao X, Vogel U, et al Subacute oral toxicity investigation of nanoparticulate and ionic silver in rats. Archives of toxicology. 2012;86(4):543–51. doi: 10.1007/s00204-011-0759-1 . 21969074 10.1007/s00204-011-0759-1

[38] Katsumiti A, Gilliland D, Arostegui I, Cajaraville MP. Mechanisms of Toxicity of Ag Nanoparticles in Comparison to Bulk and Ionic Ag on Mussel Hemocytes and Gill Cells. PloS one. 2015;10(6):e0129039 doi: 10.1371/journal.pone.0129039 ; PubMed Central PMCID: PMC4465040. 26061169 10.1371/journal.pone.0129039 PMC4465040

[39] Xiu ZM, Zhang QB, Puppala HL, Colvin VL, Alvarez PJ. Negligible particle-specific antibacterial activity of silver nanoparticles. Nano Lett. 2012;12(8):4271–5. doi: 10.1021/nl301934w . 22765771 10.1021/nl301934w

[40] De Matteis V, Malvindi MA, Galeone A, Brunetti V, De Luca E, Kote S, et al Negligible particle-specific toxicity mechanism of silver nanoparticles: the role of Ag+ ion release in the cytosol. Nanomedicine. 2015;11(3):731–9. doi: 10.1016/j.nano.2014.11.002 . 25546848 10.1016/j.nano.2014.11.002

[41] Sabella S, Carney RP, Brunetti V, Malvindi MA, Al-Juffali N, Vecchio G, et al A general mechanism for intracellular toxicity of metal-containing nanoparticles. Nanoscale. 2014;6(12):7052–61. doi: 10.1039/c4nr01234h ; PubMed Central PMCID: PMCPMC4120234. 24842463 10.1039/c4nr01234h PMC4120234

[42] Xu L, Shi C, Shao A, Li X, Cheng X, Ding R, et al Toxic responses in rat embryonic cells to silver nanoparticles and released silver ions as analyzed via gene expression profiles and transmission electron microscopy. Nanotoxicology. 2015;9(4):513–22. doi: 10.3109/17435390.2014.948942 . 25119417 10.3109/17435390.2014.948942

[43] Rahban M, Divsalar A, Saboury AA, Golestani A. Nanotoxicity and spectroscopy studies of silver nanoparticle: calf thymus DNA and k562 as targets. J Phys Chem C 2010;114(13):7.

[44] Piao MJ, Kang KA, Lee IK, Kim HS, Kim S, Choi JY, et al Silver nanoparticles induce oxidative cell damage in human liver cells through inhibition of reduced glutathione and induction of mitochondria-involved apoptosis. Toxicology letters. 2011;201(1):92–100. doi: 10.1016/j.toxlet.2010.12.010 . 21182908 10.1016/j.toxlet.2010.12.010

[45] Wise JP Sr., Goodale BC, Wise SS, Craig GA, Pongan AF, Walter RB, et al Silver nanospheres are cytotoxic and genotoxic to fish cells. Aquatic toxicology. 2010;97(1):34–41. doi: 10.1016/j.aquatox.2009.11.016 ; PubMed Central PMCID: PMC4526150. 20060603 10.1016/j.aquatox.2009.11.016 PMC4526150

[46] Gentric G, Maillet V, Paradis V, Couton D, L'Hermitte A, Panasyuk G, et al Oxidative stress promotes pathologic polyploidization in nonalcoholic fatty liver disease. The Journal of clinical investigation. 2015;125(3):981–92. doi: 10.1172/JCI73957 ; PubMed Central PMCID: PMC4362240. 25621497 10.1172/JCI73957 PMC4362240

[47] Patlolla AK, Hackett D, Tchounwou PB. Genotoxicity study of silver nanoparticles in bone marrow cells of Sprague-Dawley rats. Food and chemical toxicology: an international journal published for the British Industrial Biological Research Association. 2015 doi: 10.1016/j.fct.2015.05.005 . 26032631 10.1016/j.fct.2015.05.005 PMC4659778

[48] Zhang Y, Jiang H, Wang X. Cytidine-stabilized gold nanocluster as a fluorescence turn-on and turn-off probe for dual functional detection of Ag(+) and Hg(2+). Analytica chimica acta. 2015;870:1–7. doi: 10.1016/j.aca.2015.01.016 . 25819783 10.1016/j.aca.2015.01.016

