# Supplementary material for: Acute toxicity and genotoxicity of silver nanoparticle in rats
Source: PLoS One. 2017 Sep 27;12(9):e0185554. doi: 10.1371/journal.pone.0185554 (PMC5617228; doi:10.1371/journal.pone.0185554)
Supplement: S1 Table — (DOCX) [file pone.0185554.s001.docx]

**Supporting Information**

**Acute Toxicity and Genotoxicity of Silver Nanoparticle in** **Rats**

^1§^Hairuo Wen, ^1§^Mo Dan, ^1^Ying Yang, ^1^Jianjun Lyu, ^2^Anliang Shao, ^3^Xiang Cheng, ^2^Liang Chen, ^2*^ Liming Xu

**S1 Table. Urine analysis results of SD rats after single *i.v.* dose with AgNP (*n = 3*)**

| Time | Aminal No. | PRO | BIL | URO | SG | ERY | KET | NIT | Color |
| --- | --- | --- | --- | --- | --- | --- | --- | --- | --- |
| 0-4 h after dosing | M1 | ﹢3 | - | normal | >1.030 | ﹢3 | ﹢1 | - | red |
|  | M2 | ﹢2 | - | ﹢1 | >1.030 | ﹢3 | - | - | red |
|  | M3 | ﹢1 | - | normal | >1.030 | ﹢3 | ﹢1 | - | red |
| 24-28 h after dosing | M1 | ﹢1 | - | ﹢1 | <1.005 | +- | ﹢1 | - | pale yellow |
|  | M2 | ﹢1 | - | ﹢2 | <1.005 | ﹢3 | ﹢1 | - | pale yellow |
|  | M3 | ﹢1 | - | ﹢1 | <1.005 | ﹢1 | ﹢1 | - | pale yellow |
