# Supplementary material for: Acute toxicity and genotoxicity of silver nanoparticle in rats
Source: PLoS One. 2017 Sep 27;12(9):e0185554. doi: 10.1371/journal.pone.0185554 (PMC5617228; doi:10.1371/journal.pone.0185554)
Supplement: S1 Text — (DOCX) [file pone.0185554.s002.docx]

**Supporting Information**

**Acute Toxicity and Genotoxicity of Silver Nanoparticle in** **Rats**

^1§^Hairuo Wen, ^1§^Mo Dan, ^1^Ying Yang, ^1^Jianjun Lyu, ^2^Anliang Shao, ^3^Xiang Cheng, ^2^Liang Chen, ^2*^ Liming Xu

**S1 Text. Urine collection and analysis for rats dosed with AgNP.**

Wild type SPF rats (3 males, 8-week-old) with body weights between 279 g to 320 g purchased from Beijing Vital River Laboratory Animal Technology Co., Ltd. (Beijing, China; Animal Quality Certificate No: SCXK(Jing)2007-0001) were *i.v.* single-dosed with 5 mg/kg AgNP, and their urine samples collected within 0 to 4 hours and 24 to 28 hours after dosing were analyzed using AM-4290 Semi-Automated Urine Analyzer (ARKRAY Inc. Kyoto,Japan). Test indexes includes: protein (PRO), bilirubin (BIL), Urobilinogen (URO), specific gravity (SG), erythrocyte (ERY), ketones (KET) and nitrite (NIT), and the urine color was observed by naked eyes.
